# Early Prediction of Cognitive Deficits After Traumatic Brain Injury Based on AD-Like Patterns of Neurodegeneration

**DOI:** 10.1093/geroni/igab046.1439

**Published:** 2021-12-17

**Authors:** Andrei Irimia, Alexander Maher, Kenneth Rostowsky, Nikhil Chaudhari, Nahian Chowdhury, Elliot Jacobs, David Robles, Ammar Dharani

**Affiliations:** 1 University of Southern California, University of Southern California, California, United States; 2 University of Southern California, Los Angeles, California, United States

## Abstract

Traumatic brain injuries (TBIs) are frequently followed by persistent brain alterations and by cognitive sequalae, especially in older adults. Although mild TBI (mTBI) is a risk factor for Alzheimer’s disease (AD), the extent to which the two conditions are related remains largely unexplored. Using structural, functional and diffusion magnetic resonance imaging (MRI), we have identified AD-like post-traumatic neurodegeneration patterns that accurately prognosticate cognitive decline after geriatric mTBI. Our results indicate that these features involve cortical regions and circuitry mediating memory and executive function, and that AD neurodegeneration has key structural and functional similarities to post-traumatic neurodegradation. Using machine learning of such similarities, we have accurately forecast the severity of chronic cognitive deficits after geriatric mTBI based on acute neuroimaging measures. Our findings demonstrate that AD-like alterations in brain structure and function observed early after injury can predict post-traumatic mild cognitive impairment, which is itself strongly associated with AD risk.

